# The reversal effect of prothrombin complex concentrate (PCC), activated PCC and recombinant activated factor VII against anticoagulation of Xa inhibitor

**DOI:** 10.1186/s12959-017-0129-1

**Published:** 2017-02-20

**Authors:** Nina Haagenrud Schultz, Hoa Thi Tuyet Tran, Stine Bjørnsen, Carola Elisabeth Henriksson, Per Morten Sandset, Pål Andre Holme

**Affiliations:** 1 Research Institute of Internal Medicine, Oslo University Hospital, Box 4950, Nydalen, N-0424 Oslo, Norway; 2 Department of Haematology, Oslo University Hospital, Box 4950, Nydalen, N-0424 Oslo, Norway; 3 Department of Haematology, Akershus University Hospital, N-1478 Lørenskog, Norway; 4 Institute of Clinical Medicine, Faculty of Medicine, University of Oslo, Box 1171, Blindern, N-0318 Oslo, Norway; 5 Department of Medical Biochemistry, Oslo University Hospital, Box 4950, Nydalen, N-0424 Oslo, Norway

**Keywords:** Rivaroxaban, Reversal, Prothrombin complex concentrate, Activated prothrombin complex concentrate, Recombinant aFVIIa

## Abstract

**Background:**

An increasing number of patients are treated with direct-acting oral anticoagulants (DOACs), but the optimal way to reverse the anticoagulant effect is not known. Specific antidotes are not available and prothrombin complex concentrate (PCC), activated PCC (aPCC) and recombinant factor VIIa (rFVIIa) are variously used as reversal agents in case of a major bleeding. We aimed to determine the most effective haemostatic agent and dose to reverse the effect of rivaroxaban in blood samples from patients taking rivaroxaban for therapeutic reasons.

**Methods:**

Blood samples from rivaroxaban-treated patients (*n =* 50) were spiked with PCC, aPCC and rFVIIa at concentrations imitating 80%, 100% and 125% of suggested therapeutic doses. The reversal effect was assessed by thromboelastometry in whole blood and a thrombin generation assay (TGA) in platelet-poor plasma. Samples from healthy subjects (*n =* 40) were included as controls.

**Results:**

In thromboelastometry measurements, aPCC and rFVIIa had a superior effect to PCC in reversing the rivaroxaban-induced lenghtening of clotting time (CT). aPCC was the only haemostatic agent that shortened the CT down to below the control level. Compared to healthy controls, patients on rivaroxaban also had a prolonged lag time and decreased peak concentration, velocity index and endogenous thrombin potential (ETP) in platelet-poor plasma. aPCC reversed these parameters more effectively than rFVIIa and PCC. There were no differences in efficacy between 80%, 100% and 125% doses of aPCC.

**Conclusions:**

aPCC seems to reverse the anticoagulant effect of rivaroxaban more effectively than rFVIIa and PCC by evaluation with thromboelastometry and TGA in vitro.

## Background

The efficacy and safety of direct-acting oral anticoagulants (DOACs), including the factor Xa inhibitor rivaroxaban, in the prevention and treatment of thromboembolic disorders have been demonstrated in a number of clinical studies [1, 2]. It is documented that the associated bleeding risk is lower for rivaroxaban than for warfarin [3]. Spontaneous and trauma-induced bleeding episodes do, however, still occur in patients on DOACs [4, 5]. Large phase 3 studies have shown that the relative risk of major bleeding is 1.1% for patients taking DOACs compared to 1.8% in patients taking warfarin. Real-world data from observational studies confirm these results [6–8]. Guidelines for treatment of major bleedings on rivaroxaban are inconsistent [9, 10]. Although routines for supportive treatment, such as fluid replacement and blood transfusions, topical haemostatic measures and charcoal administration in case of recent tablet intake have been established, there is not a consensus on how to reverse the anticoagulant effect of rivaroxaban in case of major or life-threatening bleeding. A generic reversal agent of factor Xa inhibitors, andexanet alpha, has shown promising results [11], but no antidote is yet commercially available.

Three haemostatic agents have been suggested as surrogate antidotes, but the documentation on the effect and optimal dosage is limited and divergent. Four-factor prothrombin complex concentrate (PCC) is used as an antidote to warfarin, replacing coagulation factors II, VII, IX, and X in their zymogen or inactive forms. Haemophiliacs with inhibitors are treated with recombinant activated factor VII (rFVIIa) and/or activated PCC (aPCC) containing coagulation factors II, IX, and X, and FVIIa. Several studies have evaluated the reversing effect of these surrogate antidotes on haemostatic parameters in animals [12, 13] and by using blood from healthy subjects taking rivaroxaban or blood spiked with rivaroxaban ex vivo [14–20]. It has been shown that different PCCs incompletely reverse the anticoagulation effect of rivaroxaban on the thrombin generation assay (TGA) parameter endogenous thrombin potential (ETP) [21], and there is increasing evidence suggesting that aPCC and rFVIIa have a better effect [14, 17, 19]. To our knowledge, the reversing effect of those agents has not yet been studied on patients taking rivaroxaban for therapeutic reasons.

The aims of the present study were to compare PCC, aPCC and rFVIIa as surrogate antidotes in 50 patients on therapeutic rivaroxaban doses, and to find the most effective dose to reverse the anticoagulant effect of rivaroxaban in these patients.

## Methods

### Study design

This is an in vitro study where the ability of PCC, aPCC and rFVIIa to reverse the effect of rivaroxaban was tested in blood collected from patients treated with rivaroxaban.

### Participants

Fifty patients treated with therapeutic doses of rivaroxaban for various approved indications and 40 healthy controls, without previous history of vascular disease, were recruited in the study. Patients between 18 and 85 years of age who had taken rivaroxaban for more than two months were eligible. Controls were recruited from the same age group. Ongoing treatment with anti-platelet drug(s) and/or non-steroidal antiinflammatory drug(s) was an exclusion criterium for both patients and controls. All participants gave written informed consent, and the study was approved by the Norwegian regional committee for medical and health research ethics.

### Haemostatic agents and doses

We evaluated the following reversal agents in this study: 4-PCC (Cofact®, Sanquin, Amsterdam, the Netherlands), aPCC (FEIBA®, Baxter AG, Vienna, Austria) and rFVIIa (Novoseven®, NovoNordisk, Copenhagen, Denmark). The concentrations included in this study were chosen to imitate 80%, 100% and 125% of the doses suggested for clinical use in case of a major bleeding in a patient treated with a DOAC according to existing guidelines. For PCC the suggested 100% dose is 40 IU/kg, aPCC 50 IU/kg and rFVIIa 90 μg/kg [22]. The drugs were dissolved in sterile water to stock solutions of 34 IU/mL PCC, 34 IU/mL aPCC and 68 μg/mL rFVIIa. Doses of the spiked haemostatic agents were calculated assuming that an adult had 65 mL blood/kg.

### Blood collection

Blood was collected from an antecubital vein of the patients through a 21Gx19 mm butterfly needle (Vacuette® Greiner Bio-One GmbH, Kremsmunster, Austria) with minimal use of stasis. The first 2-4 mL of blood was discarded. The blood collection tubes for the measurements of thrombin generation and thromboelastometry (0,109 M citrate Monovette®, Sarstedt, Nümbrecht, Germany) were manually prefilled with Corn Trypsin Inhibitor (CTI) (Haematologic Tecnologies Incorporates, Essex Junction, VT, USA) at a final concentration of 20 μg/mL. Test tubes that were not filled completely were discarded. For anti-FXa activity measurements we used 4.5 mL Vacutainer® tubes (Becton-Dickinson, Franklin Lakes, NJ, USA) containing 0.5 mL 0.109 M buffered citrate without CTI. The blood sampling was performed at the time of presumed peak concentration of rivaroxaban in the patients, about 2 h after the drug intake.

### Preparations

For measurements of rivaroxaban concentration by an anti-FXa activity assay, citrated plasma was obtained after centrifugation for 15 min at 2000 g in RT. The supernatant was carefully collected and stored at -80 °C for 2–3 months before measurements of anti-FXa activity were performed.

Whole blood containing CTI for measurements of thromboelastometry and thrombin generation from each patient was pooled and divided into 10 aliquots of 5 mL. Haemostatic agents were added in the doses mentioned above, and one aliquot was always left untreated to represent the baseline value.

Aliquots of whole blood spiked with three different haemostatic agents at increasing concentrations and the untreated aliquot were incubated at 37 °C for 30 min. Then the samples were further subdivided. Platelet-poor plasma (PPP) was obtained by centrifugation for 13 min at 12000 g in RT, and the supernatant was carefully collected. PPP was immediately frozen and stored at -80 °C for 1–3 months before measurement of thrombin generation. The remaining whole blood was incubated at 37 °C for another 30–90 min before measurements by thromboelastometry were performed.

### Anti-FXa activity measurements

To measure rivaroxaban concentration in citrated plasma, an anti-FXa activity method calibrated for rivaroxaban was performed on STA-R Evolution® coagulometer (Diagnostica Stago S.A.S., Asnières sur Seine, France) [23] according to the manufacturer’s instructions.

### Thrombin generation assay

Thrombin generation was measured in PPP using the Calibrated Automated Thrombogram (CAT) (Diagnostica Stago, Asnière, France) with the Thrombinoscope software (Thrombinoscope BV®, Maastricht, The Netherlands) [24, 25]. PPP, supplemented with the three different reversal agents in three different concentrations, were run in triplicates. The thrombin generation parameters lag time, peak of maximum thrombin concentration, velocity index and the total amount of thrombin generated, i.e. endogenous thrombin potential (ETP), were recorded. The PPP reagent containing 5 pM TF and 4 μM phospholipids was used to initiate thrombin generation.

### Thromboelastometry

CTI-containing whole blood (with reversal agents in three different concentrations) were run in duplicates and the clotting time (CT; seconds), clot formation time (CFT; seconds), maximum velocity (MaxV; mm/s), area under curve (AUC) and maximum clot firmness (MCF; mm) were measured by ROTEM® (TEM Innovations, Munich, Germany) with low tissue factor activated ROTEM [26]. Prior to measurements, the plastic test cups were prepared with 40 μL buffer (a mixture of equal parts of buffer 1: 20 mM Hepes, 150 mM NaCl, pH 7.4 and buffer 2: 20 mM Hepes, 150 mM NaCl, 200 mM CaCl_2_, pH 7.4). Recombinant relipidated TF (Innovin®, Dade Behring, Liederbach, Germany) diluted in a total volume of 20 μL of buffer 1 was also added. To initiate the reaction, whole blood (280 μL) was added and the total volume of reagents and whole blood in each cup was 340 μL. The final TF dilution was 1:70 000, corresponding to a theoretical concentration of 0.35 pM.

### Statistical analysis

The analysis of variance (ANOVA-test) was used followed by the post-hoc test Tukey multiple comparison. Statistical calculations were performed by using SPSS version 21 (SPSS, Inc, Chicago, USA) and statistical significance was set to *p <* 0.05.

The Spearman’s rank correlation coefficient was used when assessing the relationship between rivaroxaban concentration and coagulation parameters. The data are expressed as mean value with a 95% confidence interval (CI 95%) or one standard deviation (SD).

## Results

Between October 2014 and May 2015, 50 patients treated with therapeutic doses of rivaroxaban were enrolled in the study at Akershus University Hospital. In the same time period 40 controls were included. All 50 patients used 20 mg of rivaroxaban once daily. The indications for rivaroxaban treatment were deep vein thrombosis, pulmonary embolism and atrial fibrillation. The main characteristics of patients and controls are displayed in Table 1.

**Table 1:** Characteristics of patients and controls

	Patients (*n =* 50)	Controls (*n =* 40)
Age – years	53.1 (14.9)	50.3 (12.8)
Weight – kg	87.1 (16.5)	-
Time after intake – minutes	130.1 (14.9)	-
Platelet count – x 10^9^/L	142.6 (54.7)	171.8 (54.3)
Platelet count in PRP – x 10^9^/L	146.3 (44.5)	152.4 (26.0)
Rivaroxaban dose (mg od)	20	0
Sex (female)	26 (52%)	24 (60%)
Deep vein thrombosis	21 (42%)	-
Pulmonary embolism	28 (56%)	-
Atrial fibrillation	1 (2%)	-

Values are given in mean (SD) or n (%)

### Anti-FXa activity measurements

**Fig. 1:**
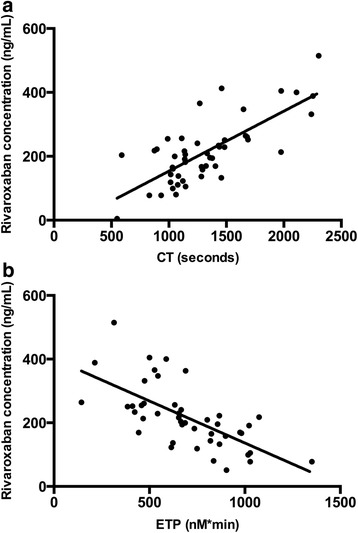
Correlation between rivaroxaban concentration and clotting time and ETP. **a**. Correlation between rivaroxaban concentration and thromboelastometry clotting time (CT) in whole blood. *R =* 0.68, *p <* 0.005, *n =* 50. **b**. Correlation between rivaroxaban concentration and endogenous thrombin potential (ETP) in platelet-poor plasma. *R =* −0.72, *p <* 0.005, *n =* 47

The mean rivaroxaban concentration in the patient group was 216.7 ng/mL (95% CI 188.2–245.3). Rivaroxaban concentrations were compared to thromboelastometry parameters in whole blood and to TGA parameters in PPP. Only data for CT and ETP are shown. The Spearman’s correlation coefficient between rivaroxaban concentration and CT was 0,68 (*p <* 0.005) (Fig. 1a). There was also a linear negative correlation between rivaroxaban levels and ETP in PPP (*r =* −0.72; *p <* 0.005) (Fig. 1b).

### Thrombin generation

**Fig. 2:**
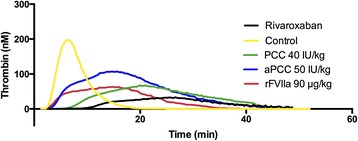
Reversal of the rivaroxaban effect by reversal agents shown in representative thrombograms. Thrombin generation was obtained by Calibrated Automated Thrombogram (CAT) on platelet-poor plasma. Thrombograms of the controls and patient samples of rivaroxaban-treated patients with and without reversal agents added. The reversal agents shown are prothrombin complex concentrate (PCC), activated PCC (aPCC) and recombinant factor VIIa (rFVIIa) in 100% dose

Rivaroxaban affected all thrombin generation parameters in PPP. Mean lag time was prolonged more than 3-fold relative to untreated controls (mean difference 6.5 min, 95% CI 5.7–7.3). Peak concentration was reduced by almost 90% (mean difference 167.1 nM, 95% CI 161.6–172.7), Velocity Index (VI) by approximately 97% (mean difference 58.2 nM/min, 95% CI 57.8–58.6) and ETP by approximately 40% (mean difference 532.9 nM*min, 95% CI 416.0–649.8) (Fig. 2).

**Fig. 3:**
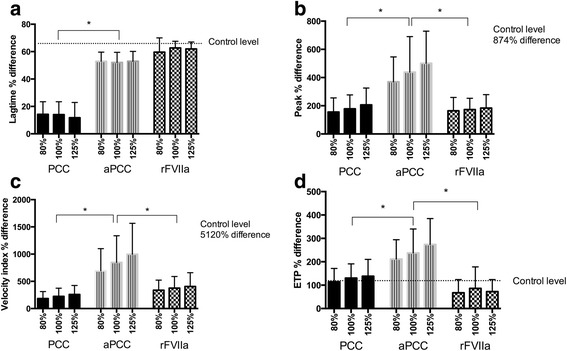
Difference in thrombin generation parameters after adding reversal agents in different doses. Differences in thrombin generation parameters are expressed as per cent difference from baseline (rivaroxaban with no haemostatic agent added). The doses of the haemostatic agents are 80%, 100% and 125% of the suggested doses, as described under study design. **a**. Lag time obtained by Calibrated Automated Thrombogram (CAT) on platelet-poor plasma (minutes); **b**. Peak height of thrombin obtained by CAT on platelet-poor plasma (nM); **c**. Velocity index obtained by CAT on platelet-poor plasma (nM/min); **d**. Endogenous Thrombin Potential (ETP) obtained by CAT on platelet-poor plasma (nM*min). Control level, the difference between the mean control value and the patient baseline values, is illustrated by the dotted line. *p < 0.005. PCC: prothrombin complex concentrate, aPCC: activated PCC, rFVIIa: recombinant factor VIIa

The haemostatic agents improved the thrombin generation parameters at varying degrees. PCC in a 100% dose did not cause a significant shortening of the lag time (mean difference 1.6 min, 95% CI 1.1–2.2). rFVIIa in 100% dose (90 μg/kg) was more effective than PCC in shortening the lag time (mean difference 6.6 min, 95% CI 5.7–7.4) but this effect was not significantly different from aPCC 100% dose which shortened lag time by 50% (mean reduction 5.5 min, 95% CI 4.7–6.3) (Fig. 3a). PCC in a 100% dose almost doubled peak concentration (mean difference 37.7 nM, 95% CI 29.5–45.9), and so did the 100% dose of rFVIIa (mean difference 36.1 nM, 95% CI 30.2–42.0). However, both those agents were less effective than aPCC 100% which caused an increase in peak concentration by 400% (mean difference 83.9 nM, 95% CI 71.4–96.4) (Fig. 3b). The velocity index (VI) was increased by 240% by PCC 100% (mean difference 3.0 nM/min, 95% CI 2.3–3.8), and by 400% by rFVIIa 100% (mean difference 6.2 nM/min, 95% CI 4.6–7.9). aPCC in the 100% dose had a significantly better reversal effect increasing VI than PCC and rFVIIa and increased VI by 800% (mean difference 12.9 nM/min, 95% CI 9.6–14.6) (Fig. 3c). The total amount of thrombin generated, ETP, was increased by the 100% dose of PCC by 130% (mean difference 802.5 nM*min, 95% CI 649.2–955.8), and by the 100% dose of rFVIIa by 80% (mean difference 452.7 nM*min, 95% CI 365.7–539.7). The 100% dose of aPCC had a more pronounced effect than PCC and rFVIIa and increased ETP by 235% (mean difference 1382.5 nM*min, 95% CI 1203.1–1561.9) (Fig. 3d).

There was not a dose–response relationship for any of the reversal agents on any of the TGA parameters. aPCC 125% of the suggested dose in clinical use reversed the TGA parameters in a similar manner to the 80% dose aPCC (lag time: *p =* 0.99, peak concentration: *p =* 1.0, velocity index: *p =* 0.99, ETP: *p =* 0.6). Furthermore the reversing effect of the lowest dose aPCC (80%) was more pronounced than the highest dose of PCC (125%) in all TGA parameters (*p <* 0.005) (Fig. 3a-d).

### Thromboelastometry

**Fig. 4:**
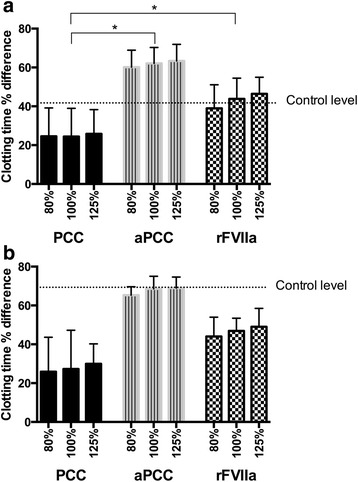
Differences in clotting time in whole blood (thromboelastometry) after adding haemostatic agents in different concentrations. Differences in clotting time expressed as per cent reduction from baseline (rivaroxaban with no haemostatic agent added). The doses of the haemostatic agents are 80%, 100% and 125% of the suggested doses, as described under study design. Control level, the difference between the mean control value and the patient baseline values, is illustrated by the dotted line. **a**. Rivaroxaban-treated patients, the whole group, *n =* 50. **b**. Subgroup with the highest rivaroxabanconcentrations (>300 ng/L), *n =* 6. *p < 0.005. PCC: prothrombin complex concentrate, aPCC: activated prothrombin complex concentrate, rFVIIa: recombinant Factor VIIa

Compared to controls, rivaroxaban doubled the CT (mean difference 674.4 s, 95% CI 527.8–820.9) (Table 2). PCC shortened the rivaroxaban-induced prolongation of CT by 25% (mean difference 386.9 s, 95% CI 263.0–510.8) and there was not a significant difference between the 80%, 100% and 125% doses (32 IU/kg, 40 IU/kg or 50 IU/kg). Adding rFVIIa caused a shortening of the CT by approximately 40% (mean difference 636.6 s, 95% CI 523.4–747.7) and aPCC shortened CT by 60% (mean difference 892.1 s, 95% CI 762.2–1022.1). There was not a significant difference between rFVIIa and aPCC in a 100% dose nor between the highest doses of these haemostatic agents. However, aPCC was the only haemostatic agent that shortened CT down below the level of the controls, and the 80% dose of aPCC was more effective than the highest dose (125%) of PCC (*p <* 0.005) (Fig. 4a)

**Table 2:** Thromboelastometry results before adding haemostatic agents

	Patients	Controls	p-value
Clotting time (CT) - sec	1379.4 (510.2)	705.8 (198.1)	<0.001
Clot formation time – sec	278.6 (157.0)	187.6 (40.1)	<0.001
Maximum clot firmness – mm	57.4 (9.7)	56.0 (5.2)	0.12
Maximum velocity – mm/s	7.0 (5.5-9.0)^a^	8.0 (7.0-10.0)^a^	0.83
Area under the curve –mm x sec	5695.3 (1103.5)	5620.4 (498.1)	0.11

Numbers are given as mean (SD) if not otherwise specified

^a^Median (interquartile range)

Treatment with rivaroxaban (Table 2) and addition of haemostatic agents (data not shown) influenced CFT in the same manner as CT. However, neither rivaroxaban (Table 2) nor the haemostatic agents (data not shown) affected the thromboelastometry parameters MCF, maxV and AUC.

### Subgroup analysis - patients with a high rivaroxaban concentration

A subgroup analysis was performed on the patients with the highest rivaroxaban concentrations, defined by a rivaroxaban concentration >300 ng/L (mean 400.6 ng/mL, 95% CI 335.3–466.0) (*n =* 6). Those patients had the longest CT in WB (mean clotting time 2425.6 s, 95% CI 1774.8–3076.3) and lowest ETP in PPP (mean ETP 531.1 nM/min, 95% CI-187.7–1249.9). We found the same pattern as in the main analysis, i.e. that aPCC increased ETP more than PCC (*p <* 0.001) and rFVIIa (*p <* 0.001). All three haemostatic agents reduced the CT in the subgroup analysis, but there was not a significant difference between the three drugs (*p =* 1.0) (Fig. 4b).

## Discussion

In the present study we evaluated the ability of three different non-specific reversal agents, PCC, aPCC, and rFVIIa, to reverse the anticoagulant effect of rivaroxaban. The TGA and thromboelastometry parameters affected by rivaroxaban were improved by all three haemostatic agents, but at various degrees. In summary, aPCC reversed rivaroxaban-induced changes in TGA (velocity index, peak height and ETP) more efficiently than PCC and rFVIIa. Also, the rivaroxaban-induced prolongation of CT in whole blood was shortened more efficiently by aPCC and rFVIIa than PCC. Adding aPCC even brought the CT to below the level of the controls, and also increased the ETP to above the control level. Furthermore, we did not find an additional effect by increasing the dose of aPCC from 80% to 125% in the assays used in this study. Our finding, i.e. that aPCC was the most effective drug, is in accordance with previous studies performed on healthy volunteers [14, 17, 19].

In this study we used the 4-factor PCC, Cofact®, which does not contain any heparin. However, to exert an antithrombotic effect, protein C and S are present in Cofact®, but this is not the case for aPCC or rFVIIa which are pure factor concentrates. How protein C and S influence the results is not known, and it is possible that their presence is the reason for the inferior effect of PCC to reverse the anticoagulant effect of rivaroxaban in this study. One possible way to find this out would be to use a 4-factor PCC which does not contain any natural anticoagulants, for example PPSB-S.D (Solvent Detergent)®(CAF-DCF, Belgium). The effect of fresh frozen plasma (FFP) has not been investigated in this study. FFP is derived from whole blood and contains all components of the coagulation cascade in physiologic concentrations, mostly in inactive forms, and is traditionally used to reverse the effect of Warfarin. Because rivaroxaban specifically inhibits FXa, replacing all factors in an inactive form is not considered as an effective treatment of a major bleeding.

The fact that PCC is widely used as a reversal agent in rivaroxaban-treated patients is partly based on findings in the study performed by Eerenberg et al. [16] where the effect of PCC as an effective reversal agent was demonstrated. In another study, Zhou et al. [13] found that PCC 50 IU/kg had effect impairing increase of cererebral haematoma induced by rivaroxaban in mice. In these studies, aPCC was not evaluated and thereafter, several studies have shown that PCC has an insufficient effect in reversing the coagulation parameters (ETP and prothrombin time in PPP) altered by rivaroxaban [21]. Furthermore, Escolar et al. and Perzborn et al. have shown that aPCC and rFVIIa had a superior effect compared to PCC in shortening the CT in whole blood (thromboelastometry) and aPCC had a better effect in improving TGA parameters in rivaroxaban-spiked samples [17, 19].

Our results were also partly in line with a study performed by Herrmann and collegues where 15 patients treated prohylactically with a low dose of rivaroxaban before orthopaedic surgery were included [27]. Here PCC and aPCC proved to be more effective reversal agents than rFVIIa, but, in contrast to our study rivaroxaban did not affect the thromboelastometry parameters CT and CFT. However, Herrmann and coauthors included patients with lower doses of rivaroxaban and a lower mean concentrations of rivaroxaban may therefore be a reasonable explanation for this difference. Another possible explanation may be different sampling conditions of whole blood (without CTI) and the use of different initiating reagents.

In our study, there was not a significant difference in efficacy between the different doses of aPCC. One explanation for the lack of dose-dependency might be that the amount of TF used to initiate the thrombin generation was too high to detect minor dose–response relationships for the reversal agents. Another reason for the lack of dose–response relationship may be that the maximum effect has already been reached at a dose of 80% aPCC. Whether this is the case in vivo, is not known. We did not test higher doses because this information in clinical practice is not considered useful. To test lower doses, however, could have given us interesting information.

There are concerns about the thrombogenicity of the haemostatic agents we have studied [28–30] and clinical data about thromboembolic complications associated with reversal of DOACs are limited. Cases have been reported where aPCC has been administrated to patients under DOAC treatment with an intracranial bleeding, and an increased risk of thrombosis was not observed [31–34]. One of the questions we asked in our study was whether a lower dose of a haemostatic agent than the guidelines recommend would be sufficient to reverse the anticoagulation effect of rivaroxaban. Because the lowest dose of aPCC (80%) was significantly more effective in reversing rivaroxaban-induced alterations in the coagulation assays than the highest doses PCC and rFVIIa, and because aPCC reversed ETP and CT to beyond the levels of the controls, one might speculate that even a lower dose of aPCC than 80% could be sufficient for reversing the rivaroxaban-induced changes. A lower dose might also reduce the risk of thromboembolic events.

Different doses of rivaroxaban to imitate an overdose situation were not included in this study. We did, however, a subgroup analysis (*n =* 6) of the patients with the highest rivaroxaban concentrations (>300 ng/mL). Those six patients also had the longest clotting time in whole blood. and the lowest ETP in PPP. Like in the main patient group we found that the reversing effect of aPCC was superior to the other reversal agents measured by ETP in PPP but we did not see a difference between the reversal agents in shortening of the CT. However, the lack of statistical difference of the post-hoc test might be due to the low number of cases in the subgroup.

A limitation of our study is that it was an in vitro study and will therefore not completely reflect the in vivo situation. In contrast to previous studies, we recruited patients taking rivaroxaban for therapeutic reasons which may have given us results closer to a real life situation. A positive impact of aPCC on the clinical outcome of an actively bleeding patient receiving rivaroxaban has been reported in several cases. This is, however, not studied in clinical trials [31–34]. It is also a general issue whether coagulation assays are predictive to assess the risk of bleeding. Only randomized clinical trials may be able to give such information.

## Conclusions

The haemostatic agent aPCC reversed rivaroxaban-induced changes in TGA parameters more efficiently than PCC and rFVIIa, and thromboelastometry parameters more efficiently than PCC in vitro. We studied three different concentrations of PCC, aPCC and rFVIIa and did not find a dose–response relationship in any of these drugs. Given the potential prothrombotic effect of these drugs, doses beyond suggestions in guidelines should be avoided.

We found a strong correlation between ETP and CT and the rivaroxaban concentration. However, future studies are needed to evaluate if these parameters can be used to identify a clinically relevant hypocoagulability or non-compliance in rivaroxaban-treated patients.

## Abbreviations

aPCC: Acivated prothrombin complex concentrate
CAT: Calibrated automated thrombogram
CFT: Clot formation time
CT: Clotting time
CTI: Corn trypsin inhibitor
DOAC: Direct-acting oral anticoagulant
ETP: Endogenous thrombin potential
MCF: Maximum clot firmness
PCC: Prothrombin complex concentrate
PPP: Platelet-poor plasma
rFVIIa: Recombinant activated Factor VII
ROTEM: Rotational Thromboelastometry
RT: Room temperature
TGA: Thrombin generation assay
VI: Velocity Index
